# How is patient activation related to healthcare service utilisation? Evidence from electronic patient records in England

**DOI:** 10.1186/s12913-021-07115-7

**Published:** 2021-11-04

**Authors:** Feifei Bu, Daisy Fancourt

**Affiliations:** Department of Behavioural Science and Health, University College London, 1-19 Torrington Place, London, WC1E 7HB UK

## Abstract

**Background:**

There is increasing awareness of the importance of patient activation (knowledge, skills, and confidence for managing one’s health and health care) among clinicians and policy makers, with emerging evidence showing higher levels of patient activation are associated with better health outcomes and experiences of health care. This study aimed to examine the association between patient activation and a wide range of specific types of healthcare service utilisation in England, including GP and non-GP primary care, elective and emergency hospital admissions, outpatient visits, and attendances at the Accident and Emergency department.

**Methods:**

Data were derived from linked electronic patient records collected by primary and secondary healthcare providers in North West London between January 2016 and November 2019. Our analyses focused on adults (18+) with a valid Patient Activation Measure (PAM). After excluding patients with missing data, we had an analytical sample of 15,877 patients. Data were analysed using negative binomial regression and logistic regression models depending on the outcome variable.

**Results:**

Patients had a mean activation score of 55.1 and a standard deviation (SD) of 17.7 (range: 0–100). They had an average of 5.4 GP visits (SD = 8.0), 26.8 non-GP visits (SD = 23.4) and 6.0 outpatient attendances (SD = 7.9) within a one-year follow-up. About 24.7% patients had at least one elective admission, 24.2% had one or more emergency admissions, and 42.3% had one or more A&E attendance within the follow-up. After accounting for a number of demographic and health factors, we found a linear (or proximately linear) association between patient activation and the number of GP visits, emergency admissions and A&E attendance, but a non-linear relationship between patient activation and the number of non-GP visits, the number of outpatient attendance and elective inpatient admission.

**Conclusions:**

This study has provided strong empirical evidence from England linking patient activation with healthcare service utilisation. It suggests the value of supporting patient activation as a potential pathway to ease the burden of healthcare system.

**Supplementary Information:**

The online version contains supplementary material available at 10.1186/s12913-021-07115-7.

## Introduction

Patient activation describes an individual’s knowledge, skills, and confidence for managing his/her health and health care [1]. Patients who display low levels of activation are typically disengaged and overwhelmed, low in health-related knowledge, with poor levels of goal-orientation and poor adherence to any preventative health regimes or treatments, seeing their health as the responsibility of their doctor. By contrast, patients who display high levels of activation act as their own health advocates, maintaining a healthy lifestyle with strong self-management skills, good knowledge of their own health, and a desire to help prevent future ill health [2]. Emerging evidence has shown that patient activation is clearly linked to a range of health-related outcomes. For example, increased patient activation has been found to predict positive changes in a number of health and self-management behaviours, such as exercise, healthy diet, treatment adherence, preventative screening and regular check-ups [2–5]. It has also been shown to be associated with better clinical indicators, such as body mass index, blood pressure, cholesterol and triglycerides [2, 6]. As a result, understanding and identifying levels of patient activation is becoming increasingly popular as part of routine care to support both the treatment plans developed for patients and to help doctors identify who could benefit from more targeted personalised care to overcome barriers relating to their activation levels [7, 8].

In addition, there is also some evidence suggesting that higher levels of patient activation are related to lower healthcare utilisation and costs [4, 6, 9–12]. For example, it was reported that highly active patients were less likely to be hospitalised and to visit emergency departments after accounting for demographic factors and disease severity [10]. A longitudinal analysis over 2 years showed that costs were significantly higher for patients whose activation level decreased and significantly lower for those who increased their activation levels, compared to patients with no change [4]. However, most previous findings to date on patient activation and healthcare utilisation have come from US data. There is a lack of evidence as to whether patient activation can also predict healthcare utilisation in other countries with different healthcare systems. It is important to explore these differing national contexts as patterns of healthcare service utilisation are likely to differ depending on factors such as whether services are free at point of contact or whether there are confounding factors such as the need for individual health insurance, which may be associated with both patient activation and health-related behaviours [13]. Additionally, studies to date have looked at relationships between patient activation and healthcare utilisation in general. But there is little detail as to whether this varies across primary or secondary care. As the measurement of patient activation becomes a more mainstream part of patient care, such knowledge could help in the planning of necessary healthcare resources both for individuals as part of their care pathways and for the healthcare sector as a whole.

To our knowledge, there is only one pervious study that examined the association between patient activation and healthcare utilisation using data from the UK. It found that patients with a higher level of activation had a lower healthcare utilisation in general and less wasteful use [14]. However, this study leaves a number of questions unanswered. First, the study used data collected from only one clinical commissioning group (CCG) in London during the pilot phrase of adopting the Patient Activation Measure (PAM) [1], which has a relatively homogeneous demographic profile of patients. There is therefore a need to understand how results might extrapolate to more diverse geographic areas and demographic groups. Secondly, the study used categorical measures of patient activation to compare how health service utilisation differed across four activation levels. However, these activation levels are broad categories, so the categorisation may obscure subtle but important differences across the whole PAM scale. To address the gaps mentioned above, the present study used routinely-collected data from eight CCGs in London, which became available after PAM was formally adopted by NHS England since 2016. We also focused on multiple different types of healthcare service utilisation (GP and non-GP primary care, elective and emergency hospital admissions, outpatient visits, and attendances to the Accident and Emergency (A&E) department) and used patient activation as a continuous measure which allowed us to explore the possibility of non-linearity across the whole spectrum of patient activation and potentially within each level.

## Methods

### Data

Data were obtained from the Whole Systems Integrated Care dashboard which links administrative records from acute, mental health and community trusts across eight CCGs, GP practices and social care data from eight boroughs in North West London. This is a demographically diverse area with regard to age, ethnicity, household income where 2.4 million people (27% of total population in London) reside. The dashboard provides real-time detailed information on how patients access and use health and social care services. We used data collected since January 2016 when PAM were introduced and patients were followed up until November 2019. Our analyses focused on adults (age 18+) who had undergone an assessment of their patient activation levels (total *N* = 19,891 patients). We excluded patients with less than one-year follow-up since their assessments (5%) and patients with missing values in any core demographic and health covariates (16%). The excluded patients due to missing covariates had similar PAM as the analytical sample (see Table S1 in the Supplement). There were 15,877 patients in our final analytical sample.

### Measures

Patient activation was measured using the 13-item PAM, a validated tool developed in the US [1]. NHS England acquired the licence for PAM and has implemented it in a number of healthcare organisations since 2016. PAM is scored on a scale from 0 to 100, with a greater value indicating a higher level of activation. PAM scores can be categorised into four activation levels, with level 1 being the least active (0–47.0), level 2 (47.1–55.1), level 3 (55.2–72.4) and level 4 the most active (72.5–100) [4].

We examined a range of outcomes within a one-year period following the initial PAM assessment for each patient. For primary care service utilisation, we looked at the number of GP consultations and other primary care contacts (e.g. general practice nurses, pharmacists etc). Both were treated as count variables derived from logged administrative records. For admitted patient care, we made a distinction between emergency and elective admissions. These were coded as binary variables indicating if patients had any emergency or elective admission respectively during the one-year follow-up. For outpatient care, we examined the number of outpatient attendances as a count variable and non-attendance as a binary variable, indicating if patients had missed any appointment. Finally, A&E attendance was coded as a binary variable, indicating if patients used A&E service within the follow-up period.

In the analyses, we controlled for a number of demographic and health covariates, including age (18–49, 50–59, 60–69, 70–79, 80–89, 90+), gender (women vs men), ethnicity (white, Asian, Black, Mixed, Other minority), area deprivation (measured by the index of multiple deprivation quantiles) and existing long-term physical and mental health conditions (no condition, 1, 2, 3, 4, 5+).

### Statistical analysis

The count outcomes (GP and non-GP visits, and outpatient attendance) were analysed using negative binomial regression models to account for over-dispersion. The binary outcomes (emergency and elective admission, outpatient non-attendance and A&E visits) were analysed using binary logistic regression models. In both set of models, we tested quadratic terms of PAM to assess the possibility of non-linearity. All analyses were adjusted for covariates identified above and conducted using Stata V15.

## Results

### Descriptive statistics

Patients included in our analysis had a mean PAM score of 55.1 and a standard deviation (SD) of 17.7. There were 27.8% patients at the lowest activation level (level 1), another 27.8% at level 2, 30.0% at level 3 and 14.3% at the highest activation level (level 4). Within the one-year follow-up, patients had an average of 5.4 GP visits (SD = 8.0) and 26.8 other non-GP primary care contacts (SD = 23.4). There were 24.7% patients having had at least one elective admission within the follow-up, and 24.2% having had one or more emergency admissions. On average, patients used outpatient services 6.0 times (SD = 7.9). About 34.3% patients had at least one outpatient appointment. Finally, 42.3% patients had one or more A&E attendance within the one-year follow-up period. For descriptive statistics of demographic and health covariates, please see Table S2 in the Supplement.

### Primary care

**Fig. 1:**
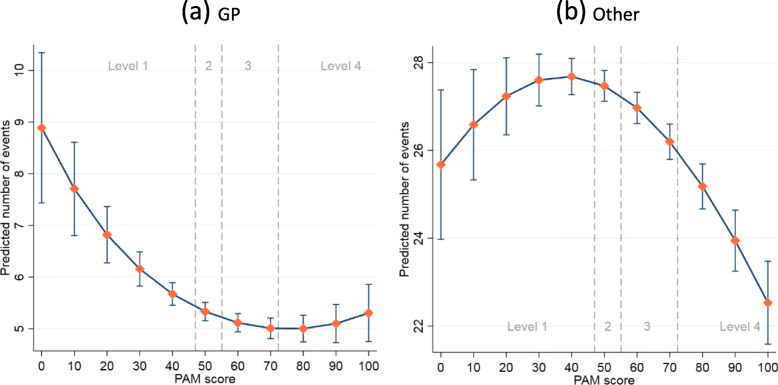
Predicted number of GP and other primary care contacts across PAM scores

Figure 1a shows the predictive margins with 95% confidence intervals (CIs) from the negative binomial regression model (see Table S3 for full results). The number of GP consultations decreased as PAM score increased in general, showing that patients with higher PAM scores consult their GP less often, especially for patients at the lowest activation level (level 1). Patients with the highest PAM scores at level 4 showed a slight increase in their number of GP consultations, but this upward trend was almost negligible.

As shown in Fig. 1b, the analysis of other primary care contacts showed a different pattern (see also Table S3). For most patients at level 1, those who with a higher PAM score used non-GP primary care services more often than their relatively less active counterparts. The relationship was reversed for patients at level 2 or above. Patients with a higher PAM score used the service less frequently.

### Admitted patient care

**Fig. 2:**
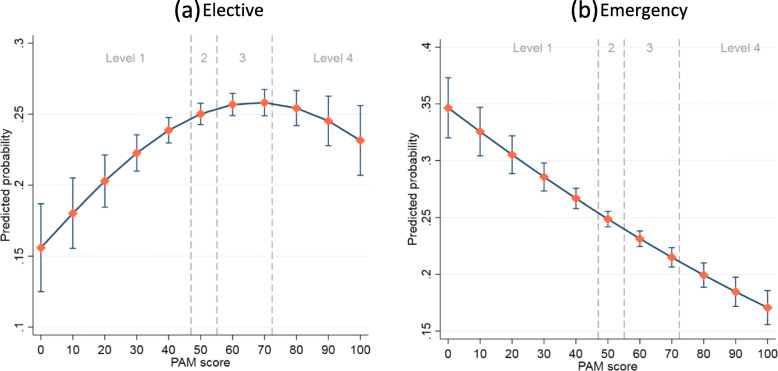
Predicted probability of elective and emergency admissions across PAM scores

The predicted probabilities of elective hospital admission with 95% CIs are presented in Fig. 2a (see Table S4 for full results). For patients at levels 1 to 3, those with higher PAM scores were more likely to have elective admissions. However, for patients at the highest activation level, the predicted probability of elective admission decreased as the PAM score increased. For emergency admissions, the predicted probability decreased consistently as PAM scores increased (Fig. 2b, see also Table S4).

### Outpatient

**Fig. 3:**
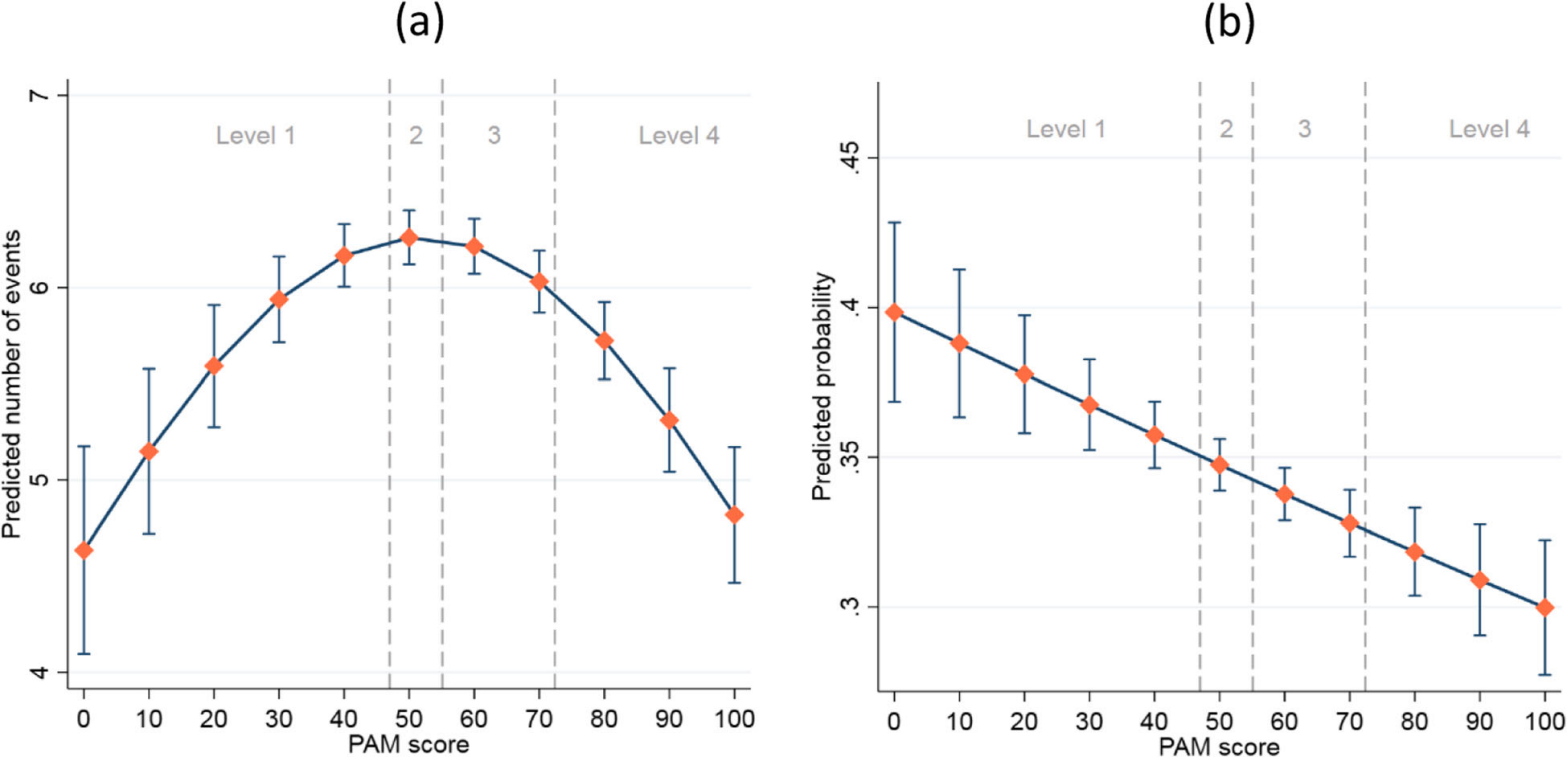
Predicted estimates of outpatients

As shown in Fig. 3a, patients at the lowest activation level had fewer outpatient appointments on average than at level 2, but the number of appointments increased as PAM score increased within level 1 (see Table S3 for full results). As PAM score increased within levels 3 and 4, the number of outpatient appointments decreased. Further, we looked at how PAM was associated with non-attendance of outpatient services. The results showed that patients with a higher PAM score were less likely to miss their outpatient appointments across all levels (Fig. 3b, see also Table S4).

### A&e

**Fig. 4:**
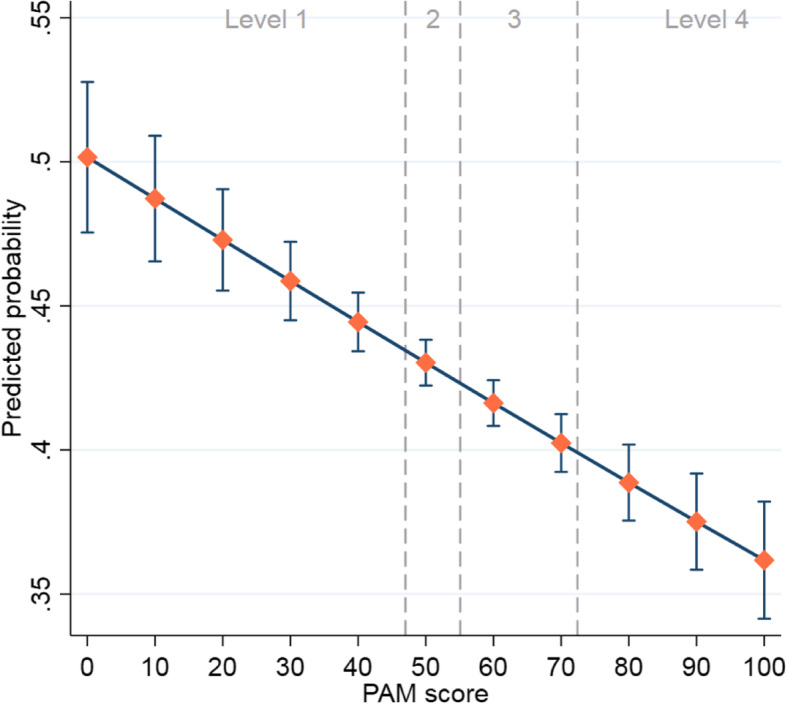
Predicted probability and 95% CIs of A&E attendance

As shown in Fig. 4, patients with a higher PAM score had a lower probabily of visiting A&E (see also Table S4). We found no evidence of nonlinearity. The predicted probability of an A&E visit decreased about 1.4% for each 10 point increase in PAM.

### Sensitivity analysis

The main results were based on complete case analysis. To investigate potential biases due to missing data, we ran sensitivity analysis using multiple imputation (chained equation, *N* = 20). The estimates of our main variable of interest, PAM, were largely the same (Table S5 and S6).

## Discussion

This study provides strong evidence linking patient activation and healthcare service utilisation using data from England, including primary (GP and non-GP) care, admitted patient care (elective and emergency admissions), outpatient and A&E attendance. However, the associations between patient activation and healthcare service utilisation varied depending on the specific service, suggesting a complex interplay between patient activation and both patient health needs and health behaviours.

As expected, patients who were more active in managing their health conditions made less use of GP services, emergency admitted patient care and A&E services compared with their less active counterparts. This is consistent with previous studies conducted in other countries [2, 4, 6, 10] and in the UK [14]. For example, in a study of over 25,000 patients in Minnesota US, it was found that the predicted probability of having an emergency department visit decreased by 1% for every 10 points increase in PAM scores [2], similar to the estimated 1.4% in our analyses. The reduction in healthcare service utilisation could be a result of patients having better health due to living healthier lifestyles (as demonstrated in previous studies of patient activation [2–4]) or delayed deterioration within existing health conditions due to better self-management of one’s health and health care. It is also possible that a higher level of patient activation contributes to a reduction in inefficient use of healthcare services, such as avoidable hospitalisation and A&E attendance. This is supported by our finding that patients who were more active were less likely to miss outpatient appointments, as well as similar findings in a previous study [14].

However, the relationship was non-linear for other healthcare services, namely non-GP consultation, elective inpatient admission and outpatient care. For these healthcare services, service utilisation decreased with patient activation within higher PAM levels, but increased as patient activation increased at lower levels. It is possible that for patients at lower activation levels, lower usage could be a result of denial or lack of recognition of symptoms that require early intervention. In contrast, an increased use in these services is a reflection of better health self-management, such as regular check-ups, preventive screening, adherence to treatments and so forth. These services are relatively less resource intensive and costly compared to other emergency healthcare services [15]. Therefore, an increased use in these services may reduce the avoidable utilisation of other more costly services, contributing to the overall efficiency of healthcare utilisation. For patients at higher activation levels, it is possible that better health management and health behaviours may render the use of these services less necessary. Therefore, while the lower utilisation of these services amongst patients at higher activation levels may not be any cause for concern, increased awareness and usage of these services at lower levels could help to reduce utilisation of more expensive emergency services in the long run.

This study has the advantage of using large scale administrative records of both primary and secondary health care and examining a wide range of healthcare services. However, it is not without limitations. First of all, this is an observational study, which is not sufficient to establish causality. We have controlled for a number of demographic and health factors, but any confounding risks by unmeasured variables cannot be ruled out. Secondly, although we examined the relationship between patient activation and subsequent service utilisations after the PAM assessment, this is a cross-sectional research design in nature. Further longitudinal investigation is recommended to understand how change in patient activation is related to change in healthcare service utilisation. Finally, our data were from London only. It remains to be explored if similar results would be found in other regions or countries in the UK.

## Conclusions

Overall, this study has provided strong empirical evidence linking patient activation with the uptake of different types of healthcare services using data from England. It suggests the importance of measuring patient activation as a way of understanding behavioural patterns relating to healthcare service utilisation. It additionally supports the value of testing whether increasing patient activation could be a potential pathway to ease the burden of healthcare system through interventions such as health coaching, peer support, self-management education and so forth [16–18]. Finally, our findings have highlighted the importance of looking at different types of healthcare services separately and making a distinction between patients with different activation levels.

## Supplementary Information

**Additional file 1: Table S1**. Descriptive statistics comparing the analytical sample with excluded patients due to missing data. **Table S2**. Descriptive statistics (*N* = 15,877). **Table S3**. Results from negative binomial regression models. **Table S4**. Results from logistic regression models. **Table S5**. Results from negative binomial regression models: multiple imputation. **Table S6**. Results from logistic regression models: multiple imputation.

## Data Availability

Data analysed during the current study are not publicly available due to data protection policy. But access can be requested from North West London Whole Systems Integrated Care (WSIC) team: https://www.nwlondonccgs.nhs.uk/.
