# Supplementary material for: How is patient activation related to healthcare service utilisation? Evidence from electronic patient records in England
Source: BMC Health Serv Res. 2021 Nov 4;21:1196. doi: 10.1186/s12913-021-07115-7 (PMC8570034; doi:10.1186/s12913-021-07115-7)
Supplement: Supplementary file 1 — Additional file 1: Table S1. Descriptive statistics comparing the analytical sample with excluded patients due to missing data. Table S2. Descriptive statistics (N = 15,877). Table S3. Results from negative binomial regression models. Table S4. Results from logistic regression models. Table S5. Results from negative binomial regression models: multiple imputation. Table S6. Results from logistic regression models: multiple imputation. [file 12913_2021_7115_MOESM1_ESM.docx]

Table S1 Descriptive statistics comparing the analytical sample with excluded patients due to missing data

|  | Frequency | Mean | Median | SD |
| --- | --- | --- | --- | --- |
| Patients with missing data (excluded) | 3,011 | 55.2 | 53.2 | 16.9 |
| Patients without missing data (analytical sample) | 15,877 | 55.1 | 53.2 | 17.7 |

Table S2 Descriptive statistics (N=15,877)

|  | Mean (SD)  % |
| --- | --- |
| PAM | 55.1 (17.7) |
| PAM level 1 | 27.8% |
| PAM level 2 | 27.8% |
| PAM level 3 | 30.0% |
| PAM level 4 | 14.3% |
| Number of GP visits | 5.4 (8.0) |
| Number of other primary care contacts | 26.8 (23.4) |
| Elective admission | 24.7% |
| Emergency admission | 24.2% |
| Number of outpatient attendance | 6.0 (7.9) |
| Non-attendance (outpatient) | 34.3% |
| A&E attendance | 42.3% |
| Women | 55.9% |
| Ethnicity: White | 36.2% |
| Ethnicity: Asian | 32.2% |
| Ethnicity: Black | 7.1% |
| Ethnicity: Mixed | 16.2% |
| Ethnicity: Other | 8.3% |
| Age: 18-49 | 10.2% |
| Age: 50-59 | 10.2% |
| Age: 60-69 | 19.4% |
| Age: 70-79 | 30.5% |
| Age: 80-89 | 24.8% |
| Age: 90+ | 5.0% |
| Index of multiple deprivation: quintile 1 | 17.2% |
| Index of multiple deprivation: quintile 2 | 31.9% |
| Index of multiple deprivation: quintile 3 | 28.8% |
| Index of multiple deprivation: quintile 4 | 16.9% |
| Index of multiple deprivation: quintile 5 | 5.3% |
| Long-term condition: 0 | 9.3% |
| Long-term condition:1 | 17.9% |
| Long-term condition: 2 | 20.6% |
| Long-term condition: 3 | 19.1% |
| Long-term condition: 4 | 14.0% |
| Long-term condition: 5+ | 19.1% |

Table S3 Results from negative binomial regression models

|  | GP  (N=15,877) | | | Non-GP  (N=15,877) | | | Outpatient  (N=15,877) | | |
| --- | --- | --- | --- | --- | --- | --- | --- | --- | --- |
|  | Coef. | SE | P | Coef. | SE | P | Coef. | SE | P |
| PAM | -0.015 | 0.003 | 0.000 | 0.004 | 0.001 | 0.000 | 0.012 | 0.002 | 0.000 |
| PAM^2^ | 0.000 | 0.000 | 0.000 | 0.000 | 0.000 | 0.000 | 0.000 | 0.000 | 0.000 |
| Women (vs. men) | -0.015 | 0.029 | 0.608 | 0.013 | 0.012 | 0.280 | -0.047 | 0.020 | 0.020 |
| Asian (vs. white) | 0.232 | 0.035 | 0.000 | -0.066 | 0.014 | 0.000 | -0.151 | 0.025 | 0.000 |
| Black (vs. white) | 0.359 | 0.059 | 0.000 | 0.009 | 0.024 | 0.721 | -0.022 | 0.042 | 0.589 |
| Mixed (vs. white) | -0.893 | 0.045 | 0.000 | 0.080 | 0.017 | 0.000 | -0.060 | 0.030 | 0.044 |
| Other (vs. white) | -0.630 | 0.057 | 0.000 | 0.082 | 0.022 | 0.000 | -0.003 | 0.039 | 0.930 |
| Age: 50-59 (vs. 18-49) | 0.111 | 0.065 | 0.086 | 0.135 | 0.026 | 0.000 | 0.122 | 0.045 | 0.007 |
| Age: 60-69 (vs. 18-49) | 0.065 | 0.057 | 0.254 | 0.202 | 0.023 | 0.000 | 0.287 | 0.040 | 0.000 |
| Age: 70-79 (vs. 18-49) | 0.107 | 0.054 | 0.046 | 0.217 | 0.022 | 0.000 | 0.395 | 0.037 | 0.000 |
| Age: 80-89 (vs. 18-49) | 0.269 | 0.055 | 0.000 | 0.216 | 0.022 | 0.000 | 0.290 | 0.039 | 0.000 |
| Age: 90+ (vs. 18-49) | 0.405 | 0.080 | 0.000 | 0.284 | 0.032 | 0.000 | 0.048 | 0.056 | 0.396 |
| IMD 2 (vs. 1) | 0.301 | 0.044 | 0.000 | -0.136 | 0.017 | 0.000 | -0.083 | 0.030 | 0.005 |
| IMD 3 (vs. 1) | 0.385 | 0.045 | 0.000 | -0.181 | 0.018 | 0.000 | -0.102 | 0.031 | 0.001 |
| IMD 4 (vs. 1) | 0.424 | 0.051 | 0.000 | -0.200 | 0.020 | 0.000 | -0.068 | 0.035 | 0.052 |
| IMD 5 (vs. 1) | 0.852 | 0.073 | 0.000 | -0.380 | 0.029 | 0.000 | -0.156 | 0.051 | 0.002 |
| Long-term condition 1 (vs. none) | 0.120 | 0.058 | 0.040 | 0.047 | 0.024 | 0.047 | 0.096 | 0.041 | 0.019 |
| Long-term condition 2 (vs. none) | 0.083 | 0.057 | 0.146 | 0.117 | 0.023 | 0.000 | 0.086 | 0.040 | 0.031 |
| Long-term condition 3 (vs. none) | 0.195 | 0.058 | 0.001 | 0.215 | 0.023 | 0.000 | 0.158 | 0.040 | 0.000 |
| Long-term condition 4 (vs. none) | 0.278 | 0.061 | 0.000 | 0.313 | 0.025 | 0.000 | 0.311 | 0.043 | 0.000 |
| Long-term condition 5 (vs. none) | 0.449 | 0.058 | 0.000 | 0.456 | 0.023 | 0.000 | 0.518 | 0.040 | 0.000 |
| _cons | 1.507 | 0.111 | 0.000 | 2.975 | 0.045 | 0.000 | 1.193 | 0.079 | 0.000 |

Table S4 Results from logistic regression models

|  | Elective inpatient care  (N=15,877) | | | Emergency inpatient care  (N=15,877) | | | Non-attendance (outpatient, N=12,279) | | | A&E  (N=15,877) | | |
| --- | --- | --- | --- | --- | --- | --- | --- | --- | --- | --- | --- | --- |
|  | Coef. | SE | P | Coef. | SE | P | Coef. | SE | P | Coef. | SE | P |
| PAM | 0.019 | 0.004 | 0.000 | -0.010 | 0.001 | 0.000 | -0.004 | 0.001 | 0.000 | -0.006 | 0.001 | 0.000 |
| PAM^2^ | 0.000 | 0.000 | 0.000 |  |  |  |  |  |  |  |  |  |
| Women (vs. men) | -0.114 | 0.038 | 0.002 | -0.099 | 0.038 | 0.010 | -0.065 | 0.039 | 0.095 | 0.007 | 0.033 | 0.831 |
| Asian (vs. white) | -0.124 | 0.047 | 0.008 | -0.201 | 0.047 | 0.000 | 0.018 | 0.048 | 0.716 | -0.147 | 0.041 | 0.000 |
| Black (vs. white) | -0.019 | 0.077 | 0.803 | -0.168 | 0.079 | 0.033 | 0.405 | 0.076 | 0.000 | -0.075 | 0.068 | 0.268 |
| Mixed (vs. white) | -0.064 | 0.056 | 0.249 | -0.244 | 0.058 | 0.000 | -0.140 | 0.059 | 0.017 | -0.076 | 0.049 | 0.120 |
| Other (vs. white) | -0.100 | 0.073 | 0.171 | -0.239 | 0.076 | 0.002 | 0.173 | 0.073 | 0.018 | -0.035 | 0.063 | 0.578 |
| Age: 50-59 (vs. 18-49) | 0.551 | 0.096 | 0.000 | -0.005 | 0.101 | 0.963 | -0.118 | 0.094 | 0.209 | -0.030 | 0.074 | 0.689 |
| Age: 60-69 (vs. 18-49) | 0.757 | 0.086 | 0.000 | 0.201 | 0.087 | 0.021 | -0.318 | 0.082 | 0.000 | -0.049 | 0.065 | 0.456 |
| Age: 70-79 (vs. 18-49) | 0.893 | 0.083 | 0.000 | 0.534 | 0.081 | 0.000 | -0.227 | 0.078 | 0.003 | 0.100 | 0.062 | 0.104 |
| Age: 80-89 (vs. 18-49) | 0.683 | 0.085 | 0.000 | 0.869 | 0.082 | 0.000 | -0.106 | 0.079 | 0.180 | 0.288 | 0.063 | 0.000 |
| Age: 90+ (vs. 18-49) | -0.021 | 0.130 | 0.870 | 1.309 | 0.105 | 0.000 | 0.007 | 0.112 | 0.951 | 0.529 | 0.091 | 0.000 |
| IMD 2 (vs. 1) | -0.094 | 0.056 | 0.092 | -0.014 | 0.057 | 0.813 | -0.214 | 0.056 | 0.000 | -0.213 | 0.049 | 0.000 |
| IMD 3 (vs. 1) | -0.122 | 0.057 | 0.034 | -0.109 | 0.059 | 0.065 | -0.405 | 0.059 | 0.000 | -0.313 | 0.050 | 0.000 |
| IMD 4 (vs. 1) | -0.081 | 0.064 | 0.208 | -0.084 | 0.066 | 0.202 | -0.360 | 0.066 | 0.000 | -0.290 | 0.057 | 0.000 |
| IMD 5 (vs. 1) | -0.068 | 0.094 | 0.467 | -0.143 | 0.095 | 0.132 | -0.629 | 0.100 | 0.000 | -0.409 | 0.083 | 0.000 |
| Long-term condition 1 (vs. none) | 0.129 | 0.082 | 0.113 | -0.203 | 0.078 | 0.010 | -0.255 | 0.083 | 0.002 | -0.074 | 0.066 | 0.265 |
| Long-term condition 2 (vs. none) | 0.065 | 0.080 | 0.417 | -0.230 | 0.077 | 0.003 | -0.250 | 0.081 | 0.002 | -0.051 | 0.065 | 0.435 |
| Long-term condition 3 (vs. none) | 0.188 | 0.080 | 0.019 | -0.160 | 0.077 | 0.039 | -0.176 | 0.082 | 0.031 | -0.020 | 0.066 | 0.756 |
| Long-term condition 4 (vs. none) | 0.334 | 0.083 | 0.000 | 0.072 | 0.080 | 0.371 | -0.072 | 0.084 | 0.391 | 0.185 | 0.069 | 0.008 |
| Long-term condition 5 (vs. none) | 0.530 | 0.079 | 0.000 | 0.372 | 0.074 | 0.000 | 0.122 | 0.079 | 0.124 | 0.414 | 0.066 | 0.000 |
| _cons | -2.396 | 0.164 | 0.000 | -0.867 | 0.123 | 0.000 | 0.154 | 0.126 | 0.220 | 0.106 | 0.102 | 0.295 |

Table S5 Results from negative binomial regression models: multiple imputation

|  | GP  (N=18,888) | | | Non-GP  (N=18,888) | | | Outpatient  (N=18,888) | | |
| --- | --- | --- | --- | --- | --- | --- | --- | --- | --- |
|  | Coef. | SE | P | Coef. | SE | P | Coef. | SE | P |
| PAM | -0.016 | 0.003 | 0.000 | 0.004 | 0.001 | 0.001 | 0.011 | 0.002 | 0.000 |
| PAM^2^ | 0.000 | 0.000 | 0.000 | 0.000 | 0.000 | 0.000 | 0.000 | 0.000 | 0.000 |

Notes: Each model controlled for the same set of covariates as the main analysis, including gender, ethnicity, age, IMD and number of long-term conditions. The estimates are omitted here.

Table S6 Results from logistic regression models: multiple imputation

|  | Elective inpatient care  (N=18,888) | | | Emergency inpatient care  (N=18,888) | | | Non-attendance (outpatient, N=14,529) | | | A&E  (N=18,888) | | |
| --- | --- | --- | --- | --- | --- | --- | --- | --- | --- | --- | --- | --- |
|  | Coef. | SE | P | Coef. | SE | P | Coef. | SE | P | Coef. | SE | P |
| PAM | 0.021 | 0.004 | 0.000 | -0.010 | 0.001 | 0.000 | -0.005 | 0.001 | 0.000 | -0.006 | 0.001 | 0.000 |
| PAM^2^ | 0.000 | 0.000 | 0.000 |  |  |  |  |  |  |  |  |  |

Notes: Each model controlled for the same set of covariates as the main analysis, including gender, ethnicity, age, IMD and number of long-term conditions. The estimates are omitted here.
